# Supplementary material for: Audio-visual modelling in a clinical setting
Source: Sci Rep. 2024 Jul 6;14:15569. doi: 10.1038/s41598-024-66160-4 (PMC11227581; doi:10.1038/s41598-024-66160-4)
Supplement: Supplementary file 1 — Supplementary Information. [file 41598_2024_66160_MOESM1_ESM.zip › Supplementary material/SupplementaryInformation.pdf]

Supplementary Information

1 Demo video for the audio-guided visual anatomy localisation application

Please refer to the attached video file 'DemoVideo.mp4' for mode details. In the video, the left sample shows the visual anatomy localisation by using our method, while the right one is the corresponding sonographer's (real human expert) eye gaze data overlaid on the same video for reference.

2 Additional ablation study about the proposed approach

In addition to the ablation study performed in the main article, here in Table A shown below, we present an additional study for the contributions of the keyword spotting (2nd row) and audio data cleaning (3rd row), over the clinical tasks of standard plane detection (columns 2 − 4) and eye-gaze saliency prediction (columns 5 − 9). The evaluation metrics follow the same as in the main article and ↑ means the higher the better, while ↓ the lower the better.

Table A: Additional ablation study on the keyword spotting and audio data cleaning.

	Precision↑	Recall↑	F1-score↑	KL↓	NSS↑	AUC↑	CC↑	SIM↑
w/o keyword spotting	76.4	76.1	75.9	3.03	2.78	0.95	0.23	0.12
w/o audio data cleaning	73.1	72.5	72.1	3.06	2.58	0.95	0.21	0.09
Ours (full)	77.2	77.3	76.8	3.00	2.80	0.95	0.23	0.12
